# WATCHMEN: a case report highlighting the closure of a bilobed left atrial appendage using two WATCHMAN-FLX devices

**DOI:** 10.1093/ehjcr/ytad607

**Published:** 2023-11-30

**Authors:** Abhinav Sood, Jagadeesh K Kalavakunta, Venu Gourineni, Vishal Gupta

**Affiliations:** Department of Interventional Cardiology, Ascension Borgess Hospital, GOC 331, 1521 Gull Road, Kalamazoo, MI 49048, USA; Department of Interventional Cardiology, Ascension Borgess Hospital, GOC 331, 1521 Gull Road, Kalamazoo, MI 49048, USA; Department of Cardiology, Ascension Borgess Hospital, Kalamazoo, MI, USA; Department of Interventional Cardiology, Ascension Borgess Hospital, GOC 331, 1521 Gull Road, Kalamazoo, MI 49048, USA

**Keywords:** Atrial fibrillation, Left atrial appendage occlusion, Watchman, Stroke, Case report

## Abstract

**Background:**

Left atrial appendage occlusion (LAAO) performed percutaneously has emerged as a widely accepted method for stroke prevention, offering a viable alternative to anticoagulation. Numerous studies have demonstrated the effectiveness and safety of this procedure. However, in certain cases, the use of a single LAAO device may not adequately achieve optimal closure due to variations in the anatomy of the left atrial appendage (LAA).

**Case summary:**

In this manuscript, we highlight the successful closure of a bilobed LAA with a large ostium utilizing two WATCHMAN™ FLX devices and using the double sheath technique. The aim was to achieve optimal closure and address the unique anatomical characteristics of the patient’s LAA.

**Discussion:**

The utilization of two LAAO devices in bilobed appendage anatomy, where a single device may not be sufficient, is possible, although it poses a challenge because of the lack of technical expertise and limited published evidence. Transoesophageal imaging can serve as a valuable tool for assessing the precise anatomy of the LAA and guide the selection and placement of the occlusion devices.

**Learning points**

- Left atrial appendage (LAA) anatomy has significant variation, and rarely, more than a single LAA occluding device may be needed for optimal closure.
- Imaging is indispensable for defining appendage anatomy. Transoesophageal or intracardiac echocardiography or cardiac computed tomography may be utilized.
- Use of two simultaneous WATCHMAN devices in a bilobed appendage with favourable anatomy is a feasible option when a single device alone does not suffice. There is currently insufficient evidence to support this technique and is not addressed in the current guidelines.

## Introduction

Atrial fibrillation (AF) independently increases the risk of ischaemic stroke 90% of the time by increasing thrombus formation in the left atrial appendage (LAA) and subsequent embolization.^1^ The Framingham Study showed a five-fold increase in stroke risk in patients with AF, even after accounting for other stroke risk factors.^2^ To address stroke prevention in patients at high bleeding risk who are unsuitable for long-term systemic anticoagulation, percutaneously implanted LAA occlusion (LAAO) devices have been approved.^3^ Clinical studies have confirmed the efficacy and safety of these devices.^3,4^ WATCHMAN™ FLX (Boston Scientific, Marlborough, MA, USA) is one of the commercially available LAAO devices used routinely for closure; however, the complexity of the LAAO procedure is determined by the significant variation in LAA shape. In rare instances, the use of a single LAAO device may not be sufficient to achieve optimal LAA closure required for reducing the risk of stroke. Incomplete closure of the LAA from the atrial cavity is an independent risk factor for thrombo-embolism.^5^ In this manuscript, we present a case of a bilobed LAA separated by a muscular ridge and with a large single ostium, necessitating the placement of two WATCHMAN™ FLX devices for complete closure.

## Summary figure

**Figure:**
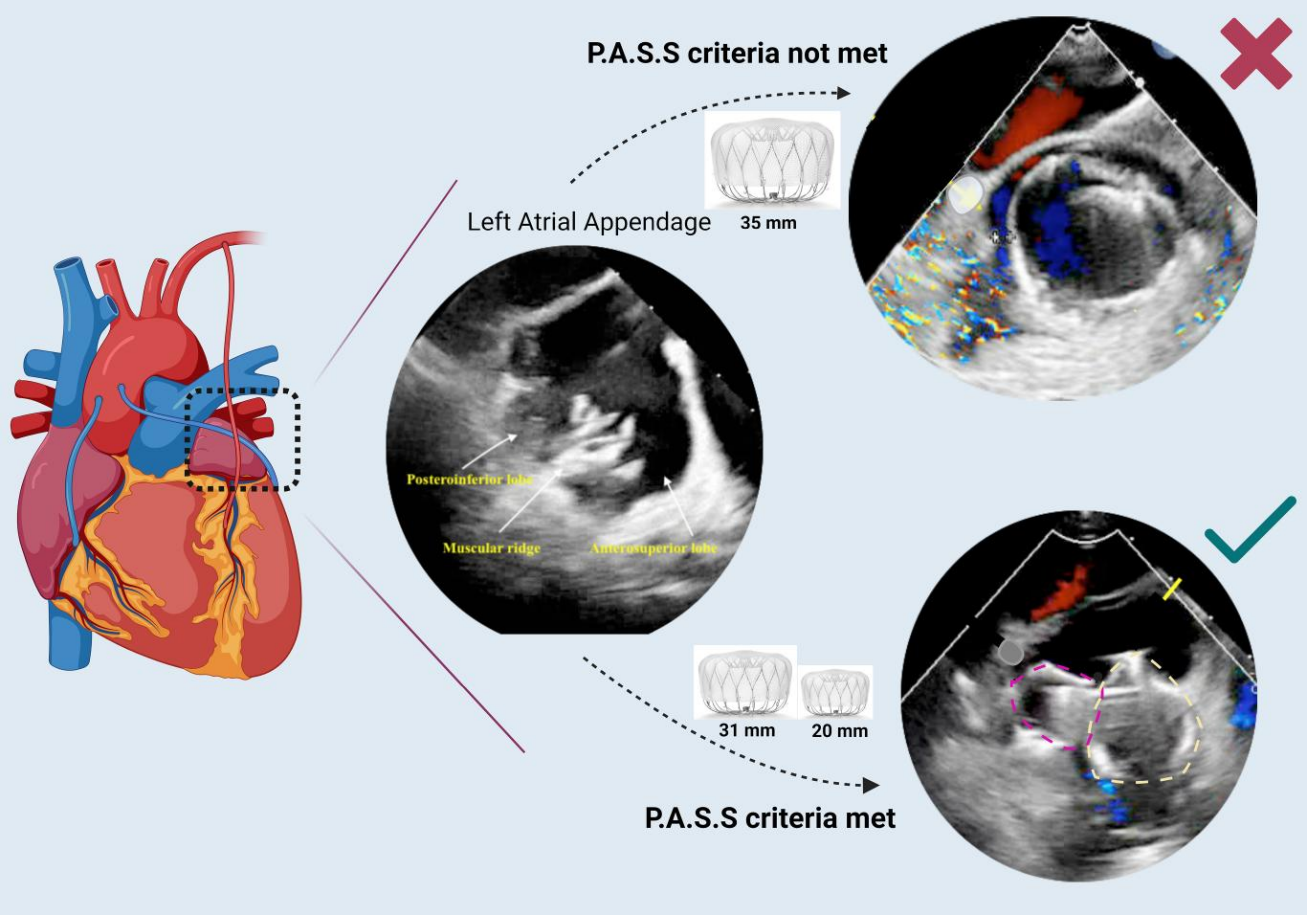


## Case presentation

A 78-year-old Caucasian gentleman with persistent AF, previous pulmonary vein isolation, coronary artery disease with remote coronary artery bypass surgery and recent percutaneous coronary intervention, and hypertension and obstructive sleep apnoea and recurrent haematuria on novel oral anticoagulation and resulting microcytic hypochromic anaemia was referred for LAAO. He was at an elevated embolic stroke and bleeding risk with a calculated CHADS_2_VASc score of 5 and a HASBLED score of 4. He was scheduled for a LAAO with a planned WATCHMAN™ FLX device with intra-procedural transoesophageal echocardiography (TEE) guidance. According to our catheterization laboratory policy, pre-procedural TEE/cardiac computed tomography (CT) is not routinely obtained for patients on anticoagulation, unless there is a high suspicion for an appendage thrombus or a previously unsuccessful occlusion attempt with a complex anatomy.

**Figure 1:**
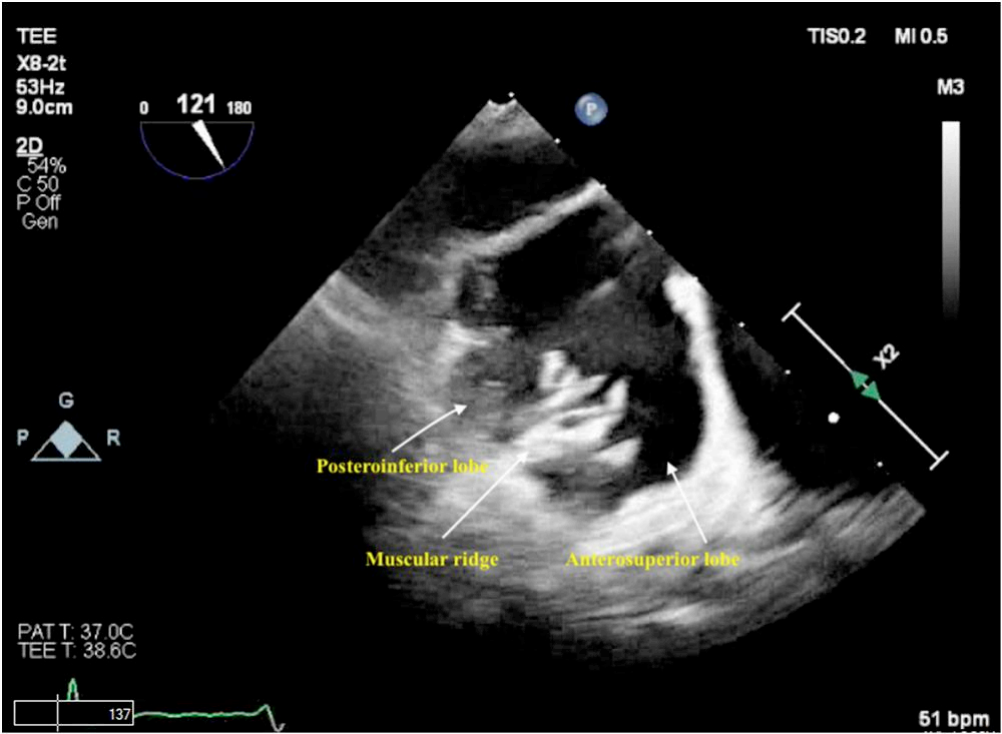
Transoesophageal echocardiogram showing the presence of a bilobed left atrial appendage separated by a muscular ridge.

**Figure 2:**
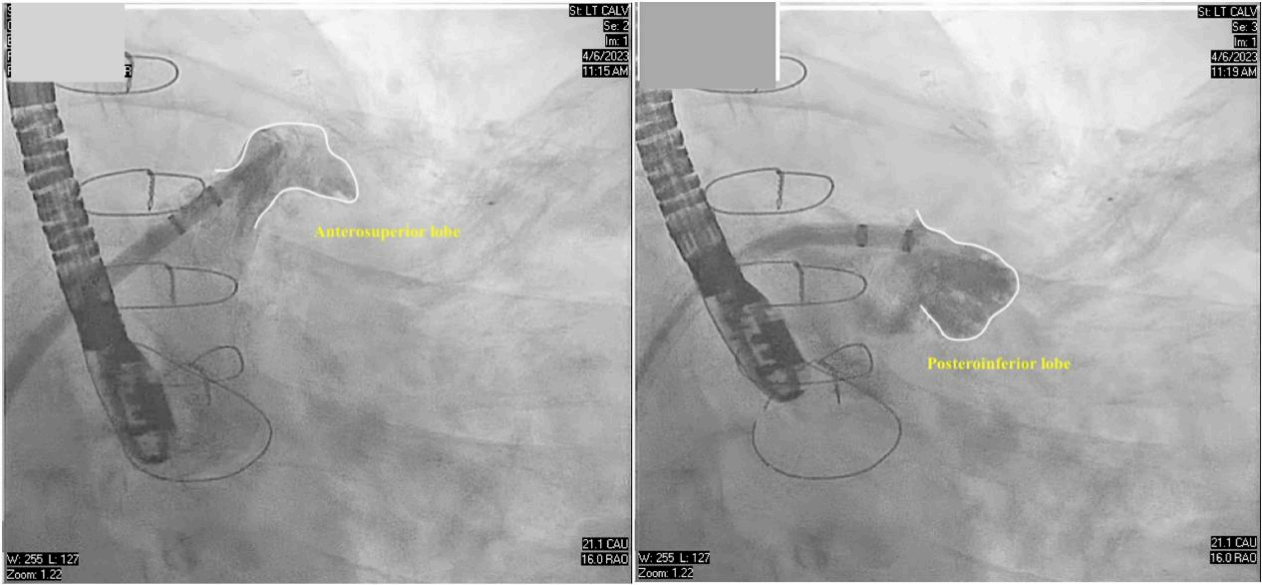
Cine angiogram in right anterior oblique caudal projection showing the separate lobes of the left atrial appendage.

**Figure 3:**
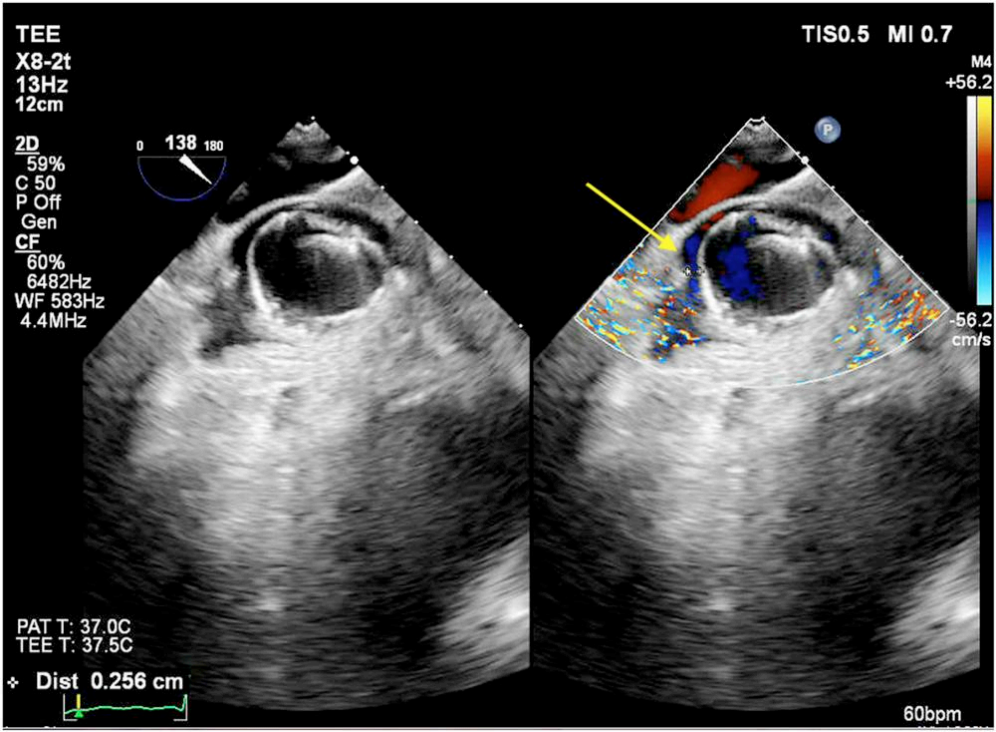
Transoesophageal echocardiogram showing incomplete occlusion of the left atrial appendage using a single 35 mm WATCHMAN device. Colour Doppler shows a >1 mm jet communicating between the anterior lobe and the left atrium.

The patient was sedated and intubated for the procedure. Ultrasound-guided right femoral vein access was obtained and an 8 French (Fr) sheath was introduced using the Seldinger technique. On TEE, the LAA measured at 30 mm × 27 mm and two lobes separated by a muscular ridge were noted. The interatrial septum was visualized and no lipomatous hypertrophy was noted. Transoesophageal echocardiography–guided trans-septal puncture (TSP) was obtained in the inferior and middle quadrant of the interatrial septum by utilizing the VersaCross™ trans-septal system using radiofrequency ablation, and a double curve trans-septal sheath with the dilator was advanced into the left atrium. We routinely utilize the VersaCross trans-septal system for all LAAO-related TSPs because of the benefit of an internally validated reduction in procedure time from venous access to left atrial access. The dilator was removed and a 6 Fr pigtail catheter was advanced over the VersaCross™ wire into the LAA and an angiogram obtained. Two separate LAA lobes separated by a muscular ridge with a single common wide ostium were noted, both on TEE and on cine-angiography (*Figures 1* and *2*). The larger of the two lobes was directed anteriorly and the other posteriorly. Closure with a single 35 mm WATCHMAN™ FLX device was attempted after accounting for 10–30% compression from the TEE-derived LAA measurements. However, the ridge prevented optimal exclusion of both lobes and biased the device anteriorly, thereby resulting in a suboptimal deployment with flow around the WATCHMAN device on colour doppler (*Figure 3*, see Supplementary material online, *Video S1*). Therefore, the 35 mm device was recaptured and removed with the sheath left in the left atrium. After a careful analysis of the TEE and the lobe angiography, it was suggested that complete closure of the appendage was possible using two devices placed sequentially and partially overlapping, such that the larger device would partially lock in the smaller device. The use of a LAMBRE device was not possible because it was not approved in the USA. Surgical ligation was deferred due to the patient’s non-preference for open surgery and elevated surgical risk stemming from a previous sternotomy and multiple comorbidities.

**Figure 4:**
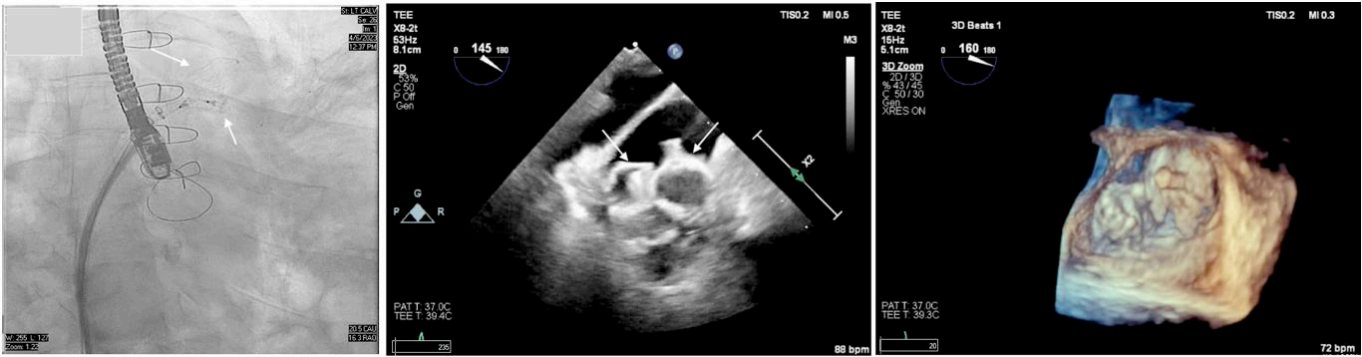
Transoesophageal echocardiogram with three-dimensional imaging and cine angiography showing successfully deployed two WATCHMAN FLX devices.

**Figure 5:**
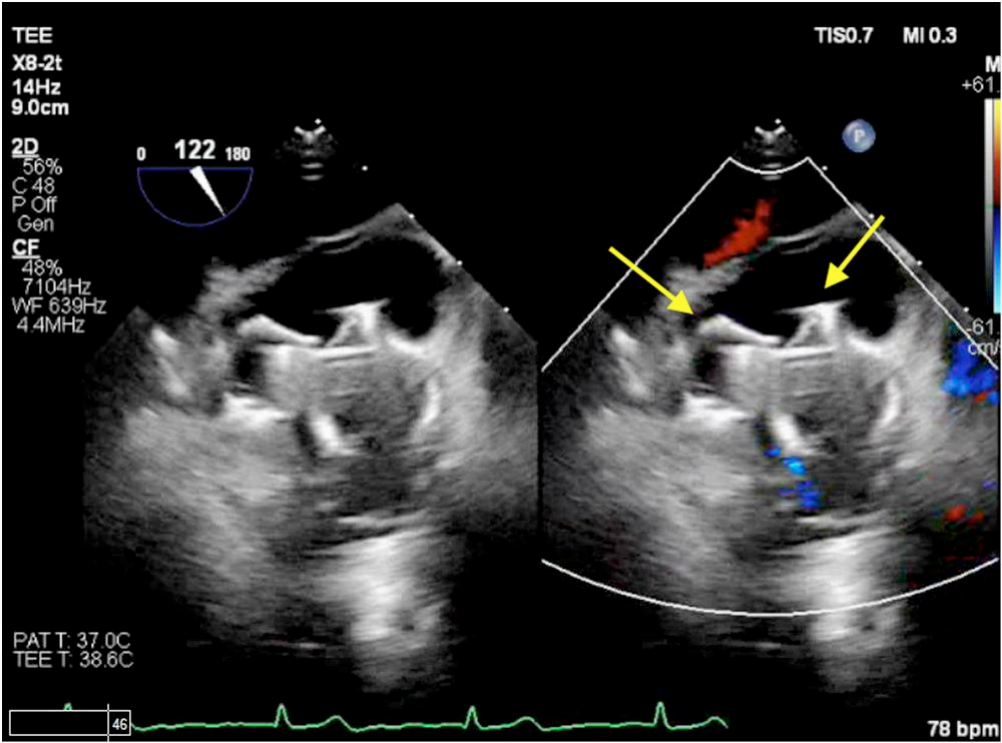
Transoesophageal echocardiogram with colour Doppler showing both WATCHMAN FLX devices (arrows) with no device thrombus and no peri-device leak.

A second TSP was obtained in the inferior and mid quadrant of the interatrial septum, using the technique described above and a second double S-curved sheath was inserted in the LAA. A 20 mm WATCHMAN™ FLX device was deployed in the smaller posterior lobe without releasing the device. A second WATCHMAN™ FLX 31 mm device was deployed in the larger anterior lobe partially covering the first one in a way to form a L-shaped configuration. Both devices were evaluated under TEE and cine-angiography, and position, anchor, size, and seal was met for both devices, and they were sequentially deployed. There was a small (<1 mm) peri-device leak at the end of the procedure and no pericardial effusion (*Figure 4*, see Supplementary material online, *Video S2*). Haemostasis was achieved and the patient was discharged the next day on home apixaban 5 mg twice daily and aspirin 81 mg once daily. He was seen in the clinic on his 45-day visit, with TEE showing well-seated devices with no peri-device leak (*Figure 5*, see Supplementary material online, *Video S3*). His apixaban was stopped and he was switched to clopidogrel 75 mg once daily and aspirin 81 mg once daily.

## Discussion

Left atrial appendage occlusion devices have been approved for preventing systemic thrombo-embolism and are endorsed by European and American guidelines.^3,4^ The shape of the LAA varies significantly between individuals, which can impact the complexity of the LAAO procedure. In rare cases, a single LAAO device may be inadequate for achieving optimal occlusion due to the anatomy of the LAA. As demonstrated in the case of our patient, the presence of a ridge with a wide neck made it challenging to exclude both lobes using a single WATCHMAN™ closure device.

We would like to emphasize the following key points derived from this case: Firstly, there are limited data regarding the use of simultaneous multiple devices for LAAO. The decision to utilize two WATCHMAN devices in our patient was based on clinical judgement rather than guided by previously published literature.^6,7^

Secondly, there are two options for delivering both devices: simultaneous delivery using two trans-septal sheaths (TSPs) or sequential delivery using the same TSP. In this case, we chose the double sheath technique to enable real-time visualization of the interaction between the two devices and to assess any residual leaks before their final release. This approach also preserved the option to recapture and change the device size if needed. The current literature reports successful outcomes with both techniques, but a direct comparison between the two is lacking.^8^

Thirdly, because of the presence of two WATCHMAN sheaths in the left atrium, surgeons should be aware of the potential interaction between the two sheaths, which depends on their proximity determined by the location of the two TSPs. In our patient, we observed minimal interaction between the sheaths, which did not affect manoeuvring or device deployment.

Finally, imaging plays a crucial role in defining appendage anatomy, and TEE, intracardiac echocardiography (ICE), and cardiac CT can be utilized. Transoesophageal echocardiography offers advantages in terms of lower cost and wider availability, making it a preferred choice for intra-operative guidance. In this case, we relied solely on TEE guidance and did not employ cardiac CT or ICE.

## Supplementary Material

ytad607_Supplementary_Data

## Data Availability

Not applicable to this case report.

## Lead author biography

Abhinav Sood is currently a fellow in interventional cardiology at the Ascension Borgess Hospital in Kalamazoo, Michigan. He obtained his bachelor’s degree in medicine and surgery from Dayanand Medical College in Punjab, India. He completed his residency in Internal Medicine from The Cleveland Clinic, Cleveland, and fellowship in Cardiology from the Mount Sinai Hospital, New York. He will be starting his advanced fellowship in structural heart disease interventions at the Ascension Borgess Hospital.
